# An original closed reduction technique for acute shoulder dislocation: the wrist-clamping and shoulder-lifting

**DOI:** 10.1186/s12245-025-00866-8

**Published:** 2025-03-26

**Authors:** Wanwu Dai, Lei Liu, Shuang Zong, Yong Zhou, Jun Zheng, Xingyan Li

**Affiliations:** 1 Department of Bone and Joint Surgery, Zhongxiang Hospital Affiliated to China Three Gorges University, Zhongxiang, 431900 Hubei China; 2 Department of Bone and Joint Surgery, The Third Affiliated Hospital of Guangxi Medical University, Nanning, 530031 Guangxi China; 3 Department of Bone and Joint Surgery, Youjiang Medical College for Nationalities Affiliated Hospital, Baise, 533000 Guangxi China

**Keywords:** Shoulder, Shoulder dislocation, Anterior shoulder dislocation, Reduction technique, Closed manual reduction, Acute

## Abstract

**Background:**

Acute anterior shoulder dislocation is one of the most common injuries in emergency medicine and orthopaedics. The aim of this study is to introduce a new closed reduction technique: the wrist-clamping and shoulder-lifting, for manual reduction of acute anterior shoulder dislocation.

**Patients and methods:**

The patient is instructed to a sitting position, the doctor hold the wrist of the arm with both hands, slowly rotated the arm to 90-degree of abduction and 60-degree of external rotation with gentle strength. After the shoulder muscles were relaxed by continuous traction, the wrist of the arm was clamped with knee joints when the arm was in 45-degree of abduction and 60-degree of external rotation. Then place hands on axilla and lift shoulder upward until the reduction is complete.

**Results:**

Thirty-six dislocated shoulders were successfully reduced with this technique, without fracture and iatrogenic neurovascular complications. No sedation, anesthesia, or intra-articular injection were used in all patients. All reduction procedures were performed by a single operator without assistance, and meantime for reduction was 3 min (range 1–8 min).

**Conclusions:**

The wrist-clamping and shoulder-lifting technique is a safe, simple, effective, gentle, fast and single-operator for anterior shoulder dislocations. Without sedation, anaesthesia, or intra-articular injection. This closed reduction technique enables orthopedists and emergency physicians to reduce the anterior shoulder dislocation smoothly and quickly, and provide a reliable and alternative reduction technique.

**Supplementary Information:**

The online version contains supplementary material available at 10.1186/s12245-025-00866-8.

## Introduction

Acute shoulder dislocation is a common sports injury in daily life, among which the anterior dislocation of the shoulder joint accounts for 95–98% [1]. Reduction should be performed as soon as possible after shoulder dislocation to avoid irreversible damage to blood vessels and nerves. At present, numerous reduction technique have been reported in the literature, and many are commonly used, such as Hippocratic method [2], the Kocher maneuver [3], Chair method [4]. The classic reduction method, Hippocrates [2], is associated with a significantly increased risk of humeral fracture and neurovascular injury. Therefore, the use of this technique is currently decreasing. In order to reduce iatrogenic neurovascular complications, we propose a new technique of reduction based on years of clinical experience. This technique makes full use of the sensitivity of the hand for reduction and avoids violence. In addition, the patient only needs to sit rather than supine position, which reduces the pain caused by position change. At the same time, it is easy to operate and only needs one person to complete it.

## Patients and methods

Thirty-six patients with anterior shoulder dislocations were successfully reduced by this technique. Clinical diagnosis of dislocation was confirmed by standard anteroposterior radiographs of the shoulder and via Y-view of the scapula and physical examination. Physical and neurologic examinations were performed before and after the reduction. All of the reductions were performed by one of our orthopedic residents. After reduction, X-ray films were reviewed to observe the glenohumeral joint alignment and whether the humerus was intact. Neurovascular injury and iatrogenic bone injury were recorded after reduction. After reduction, the chest strap was fixed for 3 weeks. All data were double-recorded by two persons, and the count data were analyzed using relevant statistical methods.

Inclusion criteria for the study were confirmed clinical diagnosis of dislocation, no neurovascular injury, simple humerus fractures of the greater tuberosity, the patient can cooperative. Exclusion criteria were posterior dislocation, previous shoulder surgery, and inability to cooperate, such as mental illness.

Mean age of the patients was 47 years, range 15 to 73 years. The right shoulder was involved in 23 of the dislocations, and 13 involved the left shoulder. The average time from the dislocation until reduction was 1.5 h, range 0.5 to 6 h. There were 26 cases of primary dislocation and 10 cases of recurrent dislocation.(Table 1).

**Table 1:** Baseline characteristics of patients managed with the Wrist-clamping and Shoulder-lifting technique

Characteristics	Data
Patients, n	36
Age, y, mean (range)	47.3(15–73)
Sex, n (%)	
Men	25(69)
Women	11(31)
Side of dislocations, n (%)	
Right	23(64)
Left	13(36)
Number of dislocations, n (%)	
Primary	26(72)
Recurrent	10(28)
Fracture-dislocation, n (%)	
No fracture	30(83)
Fracture	6(17)
Time interval, h, mean (range)	1.5(0.5-6)
Sedation, n (%)	
No sedation	36(100)
Sedation	0
Reduction result, n (%)	
Success	36(100)
Failure	0
Reduction time, min, mean (range)	3(1–8)

**Fig. 1:**
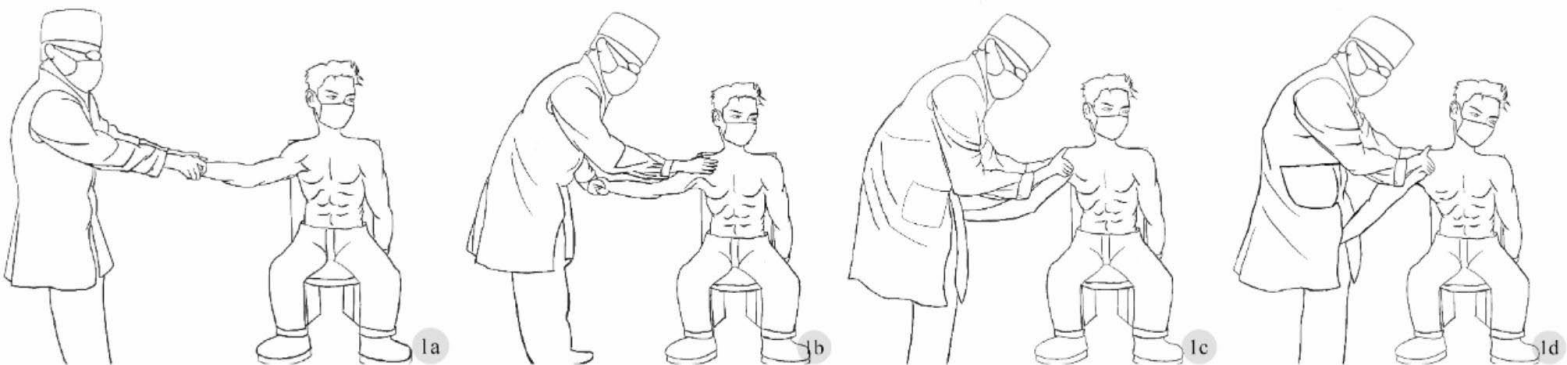
Details of the wrist-clamping and shoulder-lifting technique

Our original technique mainly consists of the following procedure: in the no anesthetic state, the patients are instructed to sit on a chair and the surgeon holds their wrist with both hands (Fig. 1a). Use the gentle force (axial traction) to slowly pull the arm to a 90-degree of abduction and 90-degree of external rotation. During continuous traction, the patient is instructed to reduce fear and relax the shoulder muscles, also can use hand to assist (Fig. 1b). Upon relaxing the patient’s shoulder muscles, the arm was positioned in a 45-degree of abduction and 90-degree of external rotation and clamped with knee joints, then using our dexterous fingers to feel the humeral head at the axilla (Fig. 1c). After determining the position of the humeral head, pull the shoulder joint upward and backward (the direction and magnitude of the force can be adjusted according to the position of the humeral head). It is indicated that the reduction is successful when feeling the bouncing sensation or sliding motion, and the deformity of the ‘square shoulder’ disappears (Fig. 1d).

After reduction, the arm was suspended with triangular towel, and the X-ray of shoulder joint was re-examined.

## Results

**Fig. 2:**
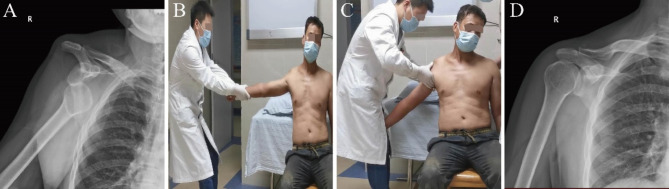
Reduction was successfully achieved through the wrist-clamping and shoulder-lifting technique without any need for sedation or anaesthesia

In this study, we evaluated the effectiveness of the wrist-clamping and shoulder-lifting technique in reduction of anterior shoulder dislocation with or without fracture of the greater tuberosity of the humerus, and with primary or recurrent dislocation. All patients had successful reduction without iatrogenic neurovascular complications. Six patients had avulsion fracture of greater tuberosity before reduction, and no new fracture was found after reduction. No sedation, anesthesia, or intra-articular injection were used in all patients. All reduction procedures were performed by a single operator without assistance, with a mean procedure time of 3 min(Fig. 2 and Video 1).

## Discussion

Shoulder dislocation is a common emergency disease. The dislocated shoulder should be reduced as soon as possible to avoid prolonged dislocation that increases the risk of vascular and nerve injury or even leads to irreversible damage. At present, there are many reduction methods reported in the literature, including Hippocratic method and traction-countertraction technique, but these reduction methods have reported the occurrence of fractures and nerve injury [5, 6].

Iatrogenic fractures are a potential threat to all reduction techniques, with the Kocher technique [6] leading to humeral neck and shaft fractures primarily due to excessive external rotation, and the Hippocratic method [2] through strong traction and leverage forces.

The glenohumeral joint is a typical ball-socket joint. The scapular glenoid is small, and the humeral head is large, and the scapular glenoid covers only 1/4 of the humeral head [7]. In terms of glenohumeral joint stability, it is mainly coordinated and compensated by the dynamic structure [8] (deltoid muscle, biceps muscle, rotator cuff muscle group) and the static structure [9, 10] (glenoid, labrum, glenohumeral ligament, joint capsule) outside the glenohumeral joint. The static structure of dishes humerus joints plays an important role to the stability of the shoulder joint in front, mainly including the jar in the ligaments, dishes humerus brachial ligament, ligament, after the jar is under brachial ligament by jar under brachial ligament toe, axillary bag, jar under brachial after ligament of beam, only guarantee that the above complete structure can avoid the occurrence of shoulder joint instability [11].

At present, the glenohumeral ligament is considered to be the most important static stable structure in front of the shoulder joint [12–14]. Some studies [10] have found that middle glenohumeral ligaments and inferior glenohumeral ligaments are in a relaxed state when abduction is 0-degree. When abduction to 45-degree, middle glenohumeral ligaments was obviously tense and elongated. At 90-degree abduction, the tension of inferior glenohumeral ligaments-anterior band increased and the ligament lengthened. In addition, it was found [15] that the glenohumeral ligament reached its maximum length at 0-degree of abduction external rotation for superior glenohumeral ligament and middle glenohumeral ligaments and at 90-degree of abduction external rotation for the anterior bundle of inferior glenohumeral ligaments. Soslowsky et al. suggested that the anterior bundle of the inferior glenohumeral ligament tensioned and limited the anterior-inferior displacement of the humeral head at 90-degree of external rotation of the shoulder [16].

Therefore, before manual reduction of shoulder dislocation, it is necessary to understand the dynamic and static biomechanical states of the shoulder joint, and to fully understand the biomechanical characteristics [17] of the shoulder joint and the state of the anterior dislocation of the shoulder joint. In the process of shoulder joint dislocation gimmick reset clinicians need to think about how to avoid the pull, rotate lips, dishes, violence and other factors, after the brachial ligament damage, further avoid resulting in a decline in late front shoulder joint stability and cause chronic shoulder joint dislocation [18].

In clinical work, through continuous summary, we found a new reduction technique: the wrist-clamping and shoulder-lifting technique. This technique uses a combination of traction, resistance, and leverage, takes full advantage of the physician’s sensitive hands and knees, and combines humanistic care (no change in position, less pain; engaging the patient and distracting). During the reduction process, the patient’s wrist was fixed with knees, and the hands were used as movable lever fulcrum. The direction and force could be changed at any time until the reduction was successful. In this technique, there may be an increased chance of fracture during traction abduction and external rotation. However, we made full use of the patient’s own resistance, thereby reducing the occurrence of fractures.

The reduction technique is mainly divided into four steps. In the first step, most patients with shoulder dislocation were admitted to the hospital on foot. In order to avoid the patient’s position change increasing the pain of the patient, we took a sitting position. Considering that the postural change from sitting to supine position will increase the patient’s pain, muscle contraction, and increase the difficulty of reduction.

The second step, the surgeon’s hands holding the patient’s wrist, with soft power pull arm slowly to outreach, outer spin 90-degree, 90-degree of brachial because at this time, after the ligaments and dishes humerus ligament can obviously under the tight, this will be conducive to dislocation of humerus head position, because of closed reduction and the basic idea is to “return to the original road”. At the same time, we can make the patient’s own resistance to traction, avoid passive violence, increase the risk of fracture, vascular and nerve injury, and even increase the risk of anterior inferior labrum injury (Bankart lesion) or Hill-Sachs lesion [19].

In the third step, when the shoulder muscles are relaxed, we can clamp the patient’s wrist with the knee joint and then use our deft fingers to feel the humeral head at the armpit.

In the fourth step, when the position of the humeral head is determined, the hand is used as a fulcrum to pull the shoulder joint up and back, and the direction and magnitude of the force can be adjusted at any time according to the position of the humeral head.

Although this complication did not occur during our reduction procedure, caution should still be exercised when performing this technique, especially in elderly patients with severe osteoporosis. Operators can reduce the risk of iatrogenic humeral fractures with the following tips:

1. When applying the traction force of abduction and external rotation, it should be slow and gradually increase the force;
2. When the patient has obvious pain and is unable to cooperate with the traction force, it indicates that there is a risk of fracture. At this time, we should pause and adjust the force and direction;
3. The hands as lever fulcrum should be in the humeral head of the axilla, not the humeral shaft.

Rapid reduction means less pain and fewer neurovascular complications. The shortest reduction time in a randomized study directed by Sayegh [20] with the Hippocratic method was 5 min, while the Kocher technique was 4 min. In our 36 cases, it was able to reduce anterior shoulder dislocation using the technique in a short time, mean 3 min (range 1–8 min).

Most current reduction techniques require the patient to lie on the bed [21–23], which will increase the patient’s pain when they move from standing to lying position. As a result, the shoulder muscle tension increases, resulting in an increase in the difficulty of reducing it, and prolongs the reduction time. Our reduction technique does not require the patient to change position, only requires the patient to sit on a chair, reducing the pain caused by the position change and allowing the patient to better participate in the reduction process, such as traction (operator) -resistance (patient).

Different from the Hippocratic method [2], Chair method [4], and Eskimo technique [24], powerful forces are easy to harm the axillary structure, resulting in vascular and nerve damage. Our reduction technique enables the use of sensitive hands (fingers) to sense the position of the humeral head, and the hand acts as a movable lever fulcrum to adjust the direction and intensity of force at any time, which avoids violent reduction and reduces the risk of fracture.

Like elbow technique [23], and unlike the Legg reduction maneuver [25] (which requires two physicians), our technique requires no assistant and can be performed by a single operator.

Our study has several limitations. First, the technique has not been applied to more patients who have not received analgesics or sedation, and the use of this method after analgesia for patients who cannot cooperate needs further research. Second, the method needs to be further compared with other methods to determine its effectiveness and safety. In addition, this technique requires a certain level of patient cooperation and has specific limitations for people with disorders of consciousness and psychosis.

## Conclusion

The wrist-clamping and shoulder-lifting technique is a safe and simple to operate, effective and gentle in action, fast and single-operator, without sedation, anaesthesia, or intra-articular injection reduction procedure for anterior shoulder dislocations. This closed reduction technique enables orthopedists and emergency physicians to reduce the anterior shoulder dislocation smoothly and quickly, and reduce the rate of reduction failure and iatrogenic complications. To provide a reliable and alternative reduction technique for orthopedic and emergency physicians.

## Electronic supplementary material

Below is the link to the electronic supplementary material.

Supplementary Material 1

## Data Availability

The datasets generated during and/or analysed during the current study are available from the corresponding author on reasonable request.
